# Bis(μ-cyclo­hexane-1,3-dicarboxyl­ato)-κ^3^ *O* ^1^:*O* ^4^,*O* ^4′^;κ^3^ *O* ^1^,*O* ^1′^:*O* ^4^-bis­[aqua­(1,10-phenanthroline-κ^2^ *N*,*N*′)zinc(II)]

**DOI:** 10.1107/S1600536809035144

**Published:** 2009-09-09

**Authors:** Mohd. Razali Rizal, Seik Weng Ng

**Affiliations:** a Department of Chemistry, University of Malaya, 50603 Kuala Lumpur, Malaysia

## Abstract

The cyclo­hexane-1,3-dicarboxyl­ate dianion in the dinuclear centrosymmetric title compound, [Zn_2_(C_8_H_10_O_4_)_2_(C_12_H_8_N_2_)_2_(H_2_O)_2_], has a chair conformation with both carboxyl­ate groups in equatorial positions. One carboxyl­ate group chelates a Zn^II^ atom, whereas the other binds through one O atom only to confer a six-coordinate status to the *N*-heterocycle-chelated water-coordinated Zn^II^ atom. Adjacent dinuclear mol­ecules are linked by O—H⋯O hydrogen bonds into a linear chain.

## Related literature

For the isostructural manganese(II) analog, see: Thirumurugan *et al.* (2006 ▶). For a review of the mol­ecular architectures of metal carboxyl­ate adducts of 2,2′-bipyridine-like ligands, see: Ye *et al.* (2003 ▶).
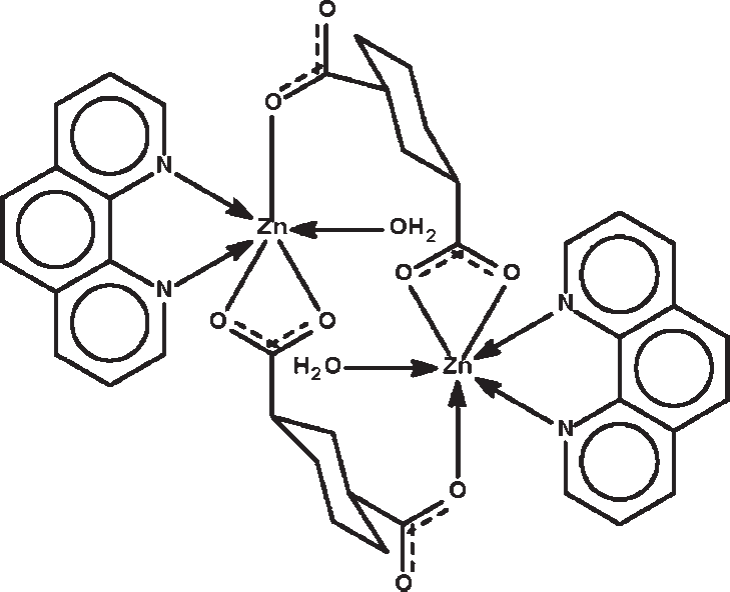


## Experimental

### 

#### Crystal data

[Zn_2_(C_8_H_10_O_4_)_2_(C_12_H_8_N_2_)_2_(H_2_O)_2_]
*M* *_r_* = 867.50
Monoclinic, 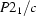

*a* = 9.6172 (2) Å
*b* = 17.4722 (5) Å
*c* = 11.4822 (3) Å
β = 104.393 (2)°
*V* = 1868.84 (8) Å^3^
*Z* = 2
Mo *K*α radiation
μ = 1.35 mm^−1^
*T* = 100 K
0.20 × 0.16 × 0.15 mm

#### Data collection

Bruker SMART APEX diffractometer
Absorption correction: multi-scan (*SADABS*; Sheldrick, 1996 ▶) *T*_min_ = 0.774, *T*_max_ = 0.823
9952 measured reflections
3289 independent reflections
2706 reflections with *I* > 2σ(*I*)
*R*_int_ = 0.032

#### Refinement

*R*[*F* ^2^ > 2σ(*F* ^2^)] = 0.069
*wR*(*F* ^2^) = 0.177
*S* = 1.09
3289 reflections
261 parameters
74 restraints
H atoms treated by a mixture of independent and constrained refinement
Δρ_max_ = 1.17 e Å^−3^
Δρ_min_ = −0.51 e Å^−3^

### 

Data collection: *APEX2* (Bruker, 2007 ▶); cell refinement: *SAINT* (Bruker, 2007 ▶); data reduction: *SAINT*; program(s) used to solve structure: *SHELXS97* (Sheldrick, 2008 ▶); program(s) used to refine structure: *SHELXL97* (Sheldrick, 2008 ▶); molecular graphics: *XSHELL* (Sheldrick, 2008 ▶); software used to prepare material for publication: *publCIF* (Westrip, 2009 ▶).

## Supplementary Material

Crystal structure: contains datablocks global, I. DOI: 10.1107/S1600536809035144/tk2534sup1.cif

Structure factors: contains datablocks I. DOI: 10.1107/S1600536809035144/tk2534Isup2.hkl

Additional supplementary materials:  crystallographic information; 3D view; checkCIF report

## Figures and Tables

**Table 1:** Hydrogen-bond geometry (Å, °)

*D*—H⋯*A*	*D*—H	H⋯*A*	*D*⋯*A*	*D*—H⋯*A*
O1*w*—H11⋯O2	0.84 (6)	1.80 (7)	2.614 (6)	162 (8)
O1*w*—H12⋯O4^i^	0.84 (6)	1.89 (3)	2.701 (6)	161 (8)

Symmetry code: (i) 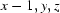.

**Fig. 1.**
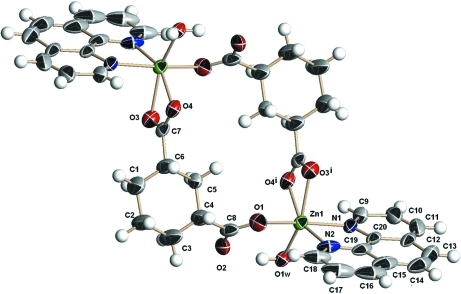
50% Probability displacement ellipsoid plot of Zn2(H2O)2(C12H8N2)2(C8H10O4)2. Hydrogen atoms are drawn as spheres of arbitrary radius. Symmetry code (i): 2-x, 1-y, 1-z.

## supplementary crystallographic information

### Experimental

Zinc acetate (0.10 g, 0.46 mmol), cyclohexane-1,3-dicarboxylic acid (mixture of *cis*- and *trans*-isomers) (0.08 g, 0.46 mmol) and 1,10-phenanthroline (0.09 g, 0.46 mmol) along with water (18 ml) were heated in a 23-ml Teflon-lined stainless-steel Parr bomb. The bomb was heated at 403 K for 3 days. The bomb was cooled to room temperature at 5 K per hour. Tiny crystals were isolated from the solution.

### Refinement

Hydrogen atoms were included in the refinement in the riding model approximation with C–H 0.95 – 1.00 Å, and with *U*(H) 1.2*U*_eq_(C). The water H-atoms were located in a difference Fourier map, and were refined with a distance restraint of O–H 0.84±0.01 Å; their displacement factors were refined.

The carbon atoms of the 1,10-phenanthroline molecule displayed somewhat elongated thermal elliposoids. As such, their anisotropic displacement factors were restrained to be nearly isotropic.

The final difference Fourier map had a peak in the vicinity of the C1 and C6 atoms.

### Crystal data

**Table:**

[Zn_2_(C_8_H_10_O_4_)_2_(C_12_H_8_N_2_)_2_(H_2_O)_2_]	*F*(000) = 896
*M**_r_* = 867.50	*D*_x_ = 1.542 Mg m^−^^3^
Monoclinic, *P*2_1_/*c*	Mo *K*α radiation, λ = 0.71073 Å
Hall symbol: -P 2ybc	Cell parameters from 2661 reflections
*a* = 9.6172 (2) Å	θ = 2.2–26.3°
*b* = 17.4722 (5) Å	µ = 1.35 mm^−^^1^
*c* = 11.4822 (3) Å	*T* = 100 K
β = 104.393 (2)°	Block, colorless
*V* = 1868.84 (8) Å^3^	0.20 × 0.16 × 0.15 mm
*Z* = 2	

### Data collection

**Table:**

Bruker SMART APEX diffractometer	3289 independent reflections
Radiation source: fine-focus sealed tube	2706 reflections with *I* > 2σ(*I*)
graphite	*R*_int_ = 0.032
ω scans	θ_max_ = 25.0°, θ_min_ = 2.2°
Absorption correction: multi-scan (*SADABS*; Sheldrick, 1996)	*h* = −11→11
*T*_min_ = 0.774, *T*_max_ = 0.823	*k* = −20→20
9952 measured reflections	*l* = −10→13

### Refinement

**Table:**

Refinement on *F*^2^	Primary atom site location: structure-invariant direct methods
Least-squares matrix: full	Secondary atom site location: difference Fourier map
*R*[*F*^2^ > 2σ(*F*^2^)] = 0.069	Hydrogen site location: inferred from neighbouring sites
*wR*(*F*^2^) = 0.177	H atoms treated by a mixture of independent and constrained refinement
*S* = 1.09	*w* = 1/[σ^2^(*F*_o_^2^) + (0.0653*P*)^2^ + 9.3251*P*] where *P* = (*F*_o_^2^ + 2*F*_c_^2^)/3
3289 reflections	(Δ/σ)_max_ = 0.001
261 parameters	Δρ_max_ = 1.17 e Å^−^^3^
74 restraints	Δρ_min_ = −0.51 e Å^−^^3^

### Fractional atomic coordinates and isotropic or equivalent isotropic displacement parameters (Å^2^)

**Table:**

	*x*	*y*	*z*	*U*_iso_*/*U*_eq_	
Zn1	0.70076 (7)	0.51170 (4)	0.71412 (7)	0.0348 (3)	
O1	0.8609 (5)	0.5855 (3)	0.7036 (5)	0.0516 (13)	
O2	0.7359 (6)	0.6719 (3)	0.5775 (5)	0.0541 (14)	
O3	1.1660 (5)	0.5852 (3)	0.3118 (4)	0.0461 (12)	
O4	1.3286 (4)	0.5440 (3)	0.4667 (4)	0.0414 (11)	
O1w	0.5440 (5)	0.5921 (3)	0.6510 (4)	0.0367 (10)	
H11	0.593 (7)	0.626 (3)	0.627 (7)	0.06 (3)*	
H12	0.471 (5)	0.572 (4)	0.606 (6)	0.05 (2)*	
N1	0.5460 (6)	0.4333 (3)	0.7566 (5)	0.0394 (13)	
N2	0.7539 (6)	0.5156 (4)	0.9034 (5)	0.0495 (16)	
C1	1.1790 (11)	0.7240 (5)	0.4694 (11)	0.085 (3)	
H1A	1.1088	0.7318	0.3912	0.102*	
H1B	1.2750	0.7386	0.4598	0.102*	
C2	1.1393 (10)	0.7760 (5)	0.5650 (11)	0.090 (4)	
H2A	1.2152	0.7730	0.6410	0.108*	
H2B	1.1322	0.8298	0.5371	0.108*	
C3	0.9953 (11)	0.7504 (5)	0.5869 (12)	0.096 (4)	
H3A	0.9754	0.7809	0.6536	0.116*	
H3B	0.9179	0.7609	0.5139	0.116*	
C4	0.9932 (9)	0.6721 (5)	0.6155 (10)	0.070 (3)	
H4	1.0652	0.6653	0.6943	0.084*	
C5	1.0389 (7)	0.6182 (4)	0.5277 (7)	0.0426 (16)	
H5A	0.9630	0.6162	0.4516	0.051*	
H5B	1.0517	0.5659	0.5620	0.051*	
C6	1.1801 (9)	0.6455 (5)	0.5022 (9)	0.064 (2)	
H6	1.2534	0.6415	0.5808	0.077*	
C7	1.2268 (6)	0.5891 (4)	0.4205 (6)	0.0369 (15)	
C8	0.8505 (7)	0.6411 (4)	0.6334 (8)	0.0499 (19)	
C9	0.4467 (8)	0.3928 (4)	0.6845 (8)	0.0502 (18)	
H9	0.4302	0.4013	0.6004	0.060*	
C10	0.3633 (9)	0.3378 (5)	0.7238 (10)	0.066 (2)	
H10	0.2932	0.3094	0.6675	0.079*	
C11	0.3834 (10)	0.3256 (5)	0.8412 (11)	0.074 (3)	
H11A	0.3270	0.2884	0.8689	0.089*	
C12	0.4866 (10)	0.3671 (5)	0.9239 (8)	0.068 (3)	
C13	0.5188 (13)	0.3622 (6)	1.0544 (11)	0.089 (3)	
H13	0.4655	0.3274	1.0899	0.107*	
C14	0.6173 (15)	0.4034 (7)	1.1242 (10)	0.094 (4)	
H14	0.6321	0.3976	1.2086	0.113*	
C15	0.7051 (12)	0.4574 (6)	1.0810 (8)	0.079 (3)	
C16	0.8171 (14)	0.5024 (7)	1.1488 (10)	0.096 (4)	
H16	0.8410	0.4975	1.2338	0.115*	
C17	0.8914 (12)	0.5518 (7)	1.0997 (10)	0.092 (4)	
H17	0.9654	0.5822	1.1484	0.110*	
C18	0.8568 (8)	0.5576 (5)	0.9736 (7)	0.065 (2)	
H18	0.9087	0.5927	0.9373	0.078*	
C19	0.6786 (9)	0.4657 (5)	0.9548 (6)	0.053 (2)	
C20	0.5701 (8)	0.4215 (4)	0.8787 (7)	0.0470 (18)	

### Atomic displacement parameters (Å^2^)

**Table:**

	*U*^11^	*U*^22^	*U*^33^	*U*^12^	*U*^13^	*U*^23^
Zn1	0.0269 (4)	0.0414 (4)	0.0374 (4)	−0.0027 (3)	0.0104 (3)	−0.0022 (3)
O1	0.031 (2)	0.049 (3)	0.076 (4)	−0.009 (2)	0.014 (2)	−0.004 (3)
O2	0.056 (3)	0.037 (3)	0.078 (4)	−0.011 (2)	0.033 (3)	−0.001 (2)
O3	0.032 (2)	0.060 (3)	0.044 (3)	0.002 (2)	0.004 (2)	0.001 (2)
O4	0.028 (2)	0.059 (3)	0.037 (3)	−0.005 (2)	0.0079 (19)	0.004 (2)
O1w	0.026 (2)	0.049 (3)	0.035 (3)	−0.002 (2)	0.008 (2)	0.002 (2)
N1	0.037 (3)	0.040 (3)	0.046 (3)	0.005 (2)	0.021 (3)	0.007 (3)
N2	0.040 (3)	0.067 (4)	0.034 (3)	0.026 (3)	−0.005 (3)	−0.017 (3)
C1	0.082 (6)	0.062 (6)	0.138 (10)	−0.014 (5)	0.079 (7)	−0.008 (6)
C2	0.080 (6)	0.057 (5)	0.164 (11)	−0.035 (5)	0.087 (7)	−0.049 (6)
C3	0.086 (7)	0.068 (6)	0.166 (11)	−0.039 (5)	0.090 (8)	−0.057 (7)
C4	0.057 (5)	0.051 (5)	0.120 (8)	−0.012 (4)	0.057 (5)	−0.016 (5)
C5	0.038 (4)	0.040 (4)	0.055 (5)	−0.008 (3)	0.019 (3)	−0.001 (3)
C6	0.055 (5)	0.056 (5)	0.098 (7)	−0.016 (4)	0.049 (5)	−0.025 (5)
C7	0.028 (3)	0.046 (4)	0.040 (4)	−0.011 (3)	0.015 (3)	−0.005 (3)
C8	0.036 (4)	0.047 (4)	0.075 (5)	−0.006 (3)	0.028 (4)	−0.022 (4)
C9	0.044 (4)	0.045 (4)	0.069 (5)	−0.008 (3)	0.028 (4)	−0.001 (3)
C10	0.054 (4)	0.049 (4)	0.109 (7)	−0.001 (3)	0.047 (5)	0.004 (4)
C11	0.065 (5)	0.050 (4)	0.126 (7)	0.019 (4)	0.059 (5)	0.028 (5)
C12	0.085 (5)	0.065 (5)	0.079 (5)	0.053 (4)	0.064 (5)	0.043 (4)
C13	0.107 (7)	0.086 (6)	0.100 (7)	0.064 (5)	0.073 (6)	0.056 (5)
C14	0.122 (7)	0.112 (7)	0.062 (6)	0.084 (6)	0.050 (6)	0.036 (5)
C15	0.091 (6)	0.090 (6)	0.055 (5)	0.067 (5)	0.020 (5)	0.009 (5)
C16	0.112 (7)	0.108 (7)	0.056 (5)	0.080 (6)	−0.003 (5)	−0.018 (5)
C17	0.077 (6)	0.102 (7)	0.071 (6)	0.055 (5)	−0.029 (5)	−0.044 (5)
C18	0.049 (4)	0.078 (5)	0.053 (4)	0.035 (4)	−0.015 (4)	−0.028 (4)
C19	0.062 (4)	0.067 (5)	0.035 (4)	0.046 (4)	0.019 (3)	0.012 (3)
C20	0.056 (4)	0.050 (4)	0.045 (4)	0.029 (3)	0.031 (3)	0.018 (3)

### Geometric parameters (Å, °)

**Table:**

Zn1—O1	2.035 (5)	C4—C5	1.521 (10)
Zn1—O3^i^	2.188 (5)	C4—C8	1.536 (9)
Zn1—O4^i^	2.247 (5)	C4—H4	1.0000
Zn1—O1w	2.056 (5)	C5—C6	1.536 (9)
Zn1—N1	2.166 (5)	C5—H5A	0.9900
Zn1—N2	2.107 (6)	C5—H5B	0.9900
O1—C8	1.250 (10)	C6—C7	1.504 (10)
O2—C8	1.251 (9)	C6—H6	1.0000
O3—C7	1.241 (8)	C9—C10	1.397 (10)
O3—Zn1^i^	2.188 (5)	C9—H9	0.9500
O4—C7	1.266 (8)	C10—C11	1.331 (14)
O4—Zn1^i^	2.247 (5)	C10—H10	0.9500
O1w—H11	0.84 (6)	C11—C12	1.394 (14)
O1w—H12	0.84 (6)	C11—H11A	0.9500
N1—C9	1.306 (9)	C12—C20	1.423 (11)
N1—C20	1.378 (9)	C12—C13	1.456 (15)
N2—C18	1.331 (10)	C13—C14	1.297 (16)
N2—C19	1.358 (11)	C13—H13	0.9500
C1—C6	1.422 (12)	C14—C15	1.434 (17)
C1—C2	1.544 (12)	C14—H14	0.9500
C1—H1A	0.9900	C15—C19	1.416 (11)
C1—H1B	0.9900	C15—C16	1.403 (16)
C2—C3	1.535 (10)	C16—C17	1.331 (17)
C2—H2A	0.9900	C16—H16	0.9500
C2—H2B	0.9900	C17—C18	1.407 (14)
C3—C4	1.408 (13)	C17—H17	0.9500
C3—H3A	0.9900	C18—H18	0.9500
C3—H3B	0.9900	C19—C20	1.412 (12)
			
O1—Zn1—O3^i^	90.3 (2)	C6—C5—H5A	109.6
O1—Zn1—O4^i^	98.2 (2)	C4—C5—H5B	109.6
O1—Zn1—O1w	92.6 (2)	C6—C5—H5B	109.6
O1—Zn1—N2	92.7 (2)	H5A—C5—H5B	108.1
O1—Zn1—N1	170.6 (2)	C1—C6—C7	116.8 (8)
O3^i^—Zn1—O4^i^	58.8 (2)	C1—C6—C5	113.7 (7)
O3^i^—Zn1—O1w	152.1 (2)	C7—C6—C5	109.3 (6)
O3^i^—Zn1—N1	90.0 (2)	C1—C6—H6	105.3
O3^i^—Zn1—N2	99.5 (2)	C7—C6—H6	105.3
O4^i^—Zn1—O1w	93.3 (2)	C5—C6—H6	105.3
O4^i^—Zn1—N1	89.9 (2)	O3—C7—O4	120.6 (6)
O4^i^—Zn1—N2	155.5 (2)	O3—C7—C6	121.6 (7)
O1w—Zn1—N1	91.6 (2)	O4—C7—C6	117.8 (7)
O1w—Zn1—N2	108.1 (2)	O2—C8—O1	125.7 (6)
N1—Zn1—N2	78.0 (2)	O2—C8—C4	118.9 (8)
C8—O1—Zn1	126.2 (5)	O1—C8—C4	115.4 (7)
C7—O3—Zn1^i^	91.9 (4)	N1—C9—C10	123.7 (8)
C7—O4—Zn1^i^	88.6 (4)	N1—C9—H9	118.1
Zn1—O1w—H11	100 (6)	C10—C9—H9	118.1
Zn1—O1w—H12	111 (5)	C11—C10—C9	119.2 (9)
H11—O1w—H12	123 (8)	C11—C10—H10	120.4
C9—N1—C20	118.4 (6)	C9—C10—H10	120.4
C9—N1—Zn1	129.4 (5)	C10—C11—C12	120.3 (8)
C20—N1—Zn1	111.9 (5)	C10—C11—H11A	119.8
C18—N2—C19	119.2 (7)	C12—C11—H11A	119.8
C18—N2—Zn1	126.6 (6)	C11—C12—C20	118.1 (8)
C19—N2—Zn1	114.1 (5)	C11—C12—C13	127.1 (9)
C6—C1—C2	111.5 (8)	C20—C12—C13	114.9 (10)
C6—C1—H1A	109.3	C14—C13—C12	122.6 (10)
C2—C1—H1A	109.3	C14—C13—H13	118.7
C6—C1—H1B	109.3	C12—C13—H13	118.7
C2—C1—H1B	109.3	C13—C14—C15	123.7 (10)
H1A—C1—H1B	108.0	C13—C14—H14	118.2
C3—C2—C1	109.7 (7)	C15—C14—H14	118.2
C3—C2—H2A	109.7	C14—C15—C19	116.7 (11)
C1—C2—H2A	109.7	C14—C15—C16	127.9 (11)
C3—C2—H2B	109.7	C19—C15—C16	115.4 (11)
C1—C2—H2B	109.7	C17—C16—C15	123.2 (10)
H2A—C2—H2B	108.2	C17—C16—H16	118.4
C4—C3—C2	112.8 (8)	C15—C16—H16	118.4
C4—C3—H3A	109.0	C16—C17—C18	117.9 (11)
C2—C3—H3A	109.0	C16—C17—H17	121.1
C4—C3—H3B	109.0	C18—C17—H17	121.1
C2—C3—H3B	109.0	N2—C18—C17	122.2 (11)
H3A—C3—H3B	107.8	N2—C18—H18	118.9
C3—C4—C5	115.1 (8)	C17—C18—H18	118.9
C3—C4—C8	116.0 (7)	N2—C19—C20	118.3 (6)
C5—C4—C8	106.6 (6)	N2—C19—C15	122.0 (9)
C3—C4—H4	106.1	C20—C19—C15	119.6 (9)
C5—C4—H4	106.1	N1—C20—C19	117.3 (6)
C8—C4—H4	106.1	N1—C20—C12	120.2 (8)
C4—C5—C6	110.4 (6)	C19—C20—C12	122.5 (8)
C4—C5—H5A	109.6		
			
O1w—Zn1—O1—C8	−26.9 (6)	C3—C4—C8—O2	29.2 (13)
N2—Zn1—O1—C8	−135.2 (6)	C5—C4—C8—O2	−100.4 (8)
O3^i^—Zn1—O1—C8	125.3 (6)	C3—C4—C8—O1	−151.5 (9)
O4^i^—Zn1—O1—C8	66.8 (6)	C5—C4—C8—O1	78.9 (9)
O1w—Zn1—N1—C9	72.9 (6)	C20—N1—C9—C10	−0.2 (10)
N2—Zn1—N1—C9	−178.9 (6)	Zn1—N1—C9—C10	172.7 (5)
O3^i^—Zn1—N1—C9	−79.2 (6)	N1—C9—C10—C11	0.8 (12)
O4^i^—Zn1—N1—C9	−20.4 (6)	C9—C10—C11—C12	−0.2 (12)
O1w—Zn1—N1—C20	−113.8 (4)	C10—C11—C12—C20	−0.9 (11)
N2—Zn1—N1—C20	−5.6 (4)	C10—C11—C12—C13	178.2 (7)
O3^i^—Zn1—N1—C20	94.1 (4)	C11—C12—C13—C14	−180.0 (9)
O4^i^—Zn1—N1—C20	152.9 (4)	C20—C12—C13—C14	−0.9 (12)
O1—Zn1—N2—C18	2.1 (6)	C12—C13—C14—C15	−0.5 (14)
O1w—Zn1—N2—C18	−91.6 (5)	C13—C14—C15—C19	1.1 (13)
N1—Zn1—N2—C18	−179.3 (6)	C13—C14—C15—C16	−177.6 (9)
O3^i^—Zn1—N2—C18	92.8 (6)	C14—C15—C16—C17	−179.2 (9)
O4^i^—Zn1—N2—C18	118.8 (6)	C19—C15—C16—C17	2.2 (12)
O1—Zn1—N2—C19	−173.6 (4)	C15—C16—C17—C18	−1.2 (14)
O1w—Zn1—N2—C19	92.7 (5)	C19—N2—C18—C17	0.5 (10)
N1—Zn1—N2—C19	5.0 (4)	Zn1—N2—C18—C17	−175.0 (5)
O3^i^—Zn1—N2—C19	−82.9 (4)	C16—C17—C18—N2	−0.2 (12)
O4^i^—Zn1—N2—C19	−56.9 (7)	C18—N2—C19—C20	−179.8 (6)
C6—C1—C2—C3	−55.1 (14)	Zn1—N2—C19—C20	−3.8 (7)
C1—C2—C3—C4	53.5 (14)	C18—N2—C19—C15	0.6 (9)
C2—C3—C4—C5	−52.1 (13)	Zn1—N2—C19—C15	176.6 (5)
C2—C3—C4—C8	−177.5 (9)	C14—C15—C19—N2	179.3 (6)
C3—C4—C5—C6	48.8 (11)	C16—C15—C19—N2	−1.8 (10)
C8—C4—C5—C6	178.9 (8)	C14—C15—C19—C20	−0.2 (10)
C2—C1—C6—C7	−176.1 (8)	C16—C15—C19—C20	178.6 (6)
C2—C1—C6—C5	55.2 (12)	C9—N1—C20—C19	179.7 (6)
C4—C5—C6—C1	−50.4 (11)	Zn1—N1—C20—C19	5.5 (7)
C4—C5—C6—C7	177.0 (8)	C9—N1—C20—C12	−0.9 (9)
Zn1^i^—O3—C7—O4	1.5 (6)	Zn1—N1—C20—C12	−175.0 (5)
Zn1^i^—O3—C7—C6	−176.8 (5)	N2—C19—C20—N1	−1.3 (9)
Zn1^i^—O4—C7—O3	−1.4 (6)	C15—C19—C20—N1	178.3 (6)
Zn1^i^—O4—C7—C6	176.9 (5)	N2—C19—C20—C12	179.3 (6)
C1—C6—C7—O3	−56.5 (10)	C15—C19—C20—C12	−1.2 (10)
C5—C6—C7—O3	74.4 (9)	C11—C12—C20—N1	1.4 (9)
C1—C6—C7—O4	125.2 (9)	C13—C12—C20—N1	−177.7 (6)
C5—C6—C7—O4	−103.9 (8)	C11—C12—C20—C19	−179.1 (6)
Zn1—O1—C8—O2	17.5 (11)	C13—C12—C20—C19	1.7 (9)
Zn1—O1—C8—C4	−161.7 (5)		

Symmetry codes: (i) −*x*+2, −*y*+1, −*z*+1.

### Hydrogen-bond geometry (Å, °)

**Table:**

*D*—H···*A*	*D*—H	H···*A*	*D*···*A*	*D*—H···*A*
O1w—H11···O2	0.84 (6)	1.80 (7)	2.614 (6)	162 (8)
O1w—H12···O4^ii^	0.84 (6)	1.89 (3)	2.701 (6)	161 (8)

Symmetry codes: (ii) *x*−1, *y*, *z*.
